# Size-dependent penetration of nanoemulsions into epidermis and hair follicles: implications for transdermal delivery and immunization

**DOI:** 10.18632/oncotarget.17130

**Published:** 2017-04-16

**Authors:** Rui Su, Wufa Fan, Qin Yu, Xiaochun Dong, Jianping Qi, Quangang Zhu, Weili Zhao, Wei Wu, Zhongjian Chen, Ye Li, Yi Lu

**Affiliations:** ^1^ Shaanxi University of Chinese Medicine, Xianyang, P.R. China; ^2^ School of Pharmacy, Fudan University, Key Laboratory of Smart Drug Delivery of MOE and PLA, Shanghai, P.R.China; ^3^ Shaanxi Academy of Traditional Chinese Medicine, Xi'an, P.R. China; ^4^ Shanghai Dermatology Hospital, Shanghai, P.R. China

**Keywords:** nanoemulsions, transdermal delivery, immunization, aggregation-caused quenching fluorescence, particle size

## Abstract

Nanoemulsions have been widely applied to dermal and transdermal drug delivery. However, whether and to what depth the integral nanoemulsions can permeate into the skin is not fully understood. In this study, an environment-responsive dye, P4, was loaded into nanoemulsions to track the transdermal translocation of the nanocarriers, while coumarin-6 was embedded to represent the cargoes. Particle size has great effects on the transdermal transportation of nanoemulsions. Integral nanoemulsions with particle size of 80 nm can diffuse into but not penetrate the viable epidermis. Instead, these nanoemulsions can efficiently fill the whole hair follicle canals and reach as deep as 588 μm underneath the dermal surfaces. The cargos are released from the nanoemulsions and diffuse into the surrounding dermal tissues. On the contrary, big nanoemulsions, with mean particle size of 500 nm, cannot penetrate the stratum corneum and can only migrate along the hair follicle canals. Nanoemulsions with median size, e.g. 200 nm, show moderate transdermal permeation effects among the three-size nanoemulsions. In addition, colocalization between nanoemulsions and immunofluorescence labeled antigen-presenting cells was observed in the epidermis and the hair follicles, implying possible capture of nanoemulsions by these cells. In conclusion, nanoemulsions are advantageous for transdermal delivery and potential in transcutaneous immunization.

## INTRODUCTION

Skin is an attractive route for both local and systemic delivery of drugs [1–4]. However, the stratum corneum (SC), a ‘bricks and mortar' structure, prevents entry of harmful xenobiotics as well as most therapeutic compounds into the body [5]. The lipophilic and intact SC layers allow only small (< 500 Da) and moderately lipophilic molecules (logP 1-3) to diffuse through passively [6]. Irrespective of dermal or transdermal delivery, it is essential to overcome the SC barriers, leading to emerging of a series of passive and active strategies. The passive strategies employ penetration enhancers [7–9], nanocarriers [10–13] and prodrugs [14, 15] to improve dermal permeation, whilst the active strategies damage the SC barriers using physical tools, such as tape stripping, microneedles, iontophoresis, electroporation, sonophoresis and jet injection [2, 16]. Considering the availability of instruments and patient compliance, nanocarriers are preferable to the physical tools.

Besides delivery of chemical drugs, skin has been increasingly realized as an outstanding route to stimulate robust immunity [17]. Because of continuous exposure to external pathogens, skin develops competent immune systems to elicit immune responses in case the physical barrier is damaged due to nicks and cuts. Both Langerhans cells that reside in the viable epidermis and dendritic cells that locate in the dermal layer are potent antigen-presenting cells (APCs), which capture and present pathogen-derived antigens to induce adaptive immune responses [17–19]. Due to the dense population of APCs in the skin, transcutaneous immunization (TCI) is endowed with superior immunogenicity as well as analgesia compared with systemic and intramuscular immunization [20]. Similarly, overcoming the SC barriers is full of challenges, especially for biomacromolecules such as vaccines. Polymeric nanoparticles were recently applied to encapsulate immunologic active materials for TCI [21–25]. However, due to poor dermal permeation into the rigid structure, tape stripping or iontophoresis should be combined to facilitate transdermal delivery [21–23, 26]. Besides, adjuvants are necessary to be combined with nanoparticles to generate efficient immune responses [24].

Nanoemulsions are aqueous dispersed ultrafine oil droplets stabilized by surfactants and co-surfactants, with a normal size less than 500 nm [27]. It should be noted that nanoemulsions are different to microemulsions, because the latter are referred to thermodynamically stable isotropic liquids formed by mixing oil, water, and surfactants together [28]. Due to the fluidic microstructure, high solubilizing capacity for lipophilic drugs and excellent skin affinity, nanoemulsions show superior transdermal efficiency to rigid nanoparticles [3, 29]. In addition, the compositions of nanoemulsions, i.e. oils, surfactants and co-surfactants, are frequently applied as penetration enhancers, improving skin permeability by altering lipid structure and fluidity of SC [30, 31]. Several topical preparations based on nanoemulsions have been approved for clinical use, e.g. Estrasorb^®^ (estradiol), Flexogan^®^ (methy salicylate), Oxalgin nanogel^®^ (diclofenac sodium) and Ameluz^®^ (5-amino levulinic acid). Nanoemulsions were utilized as antigen and immunopotentiator carrier for TCI recently [32]. However, *in vivo* fate of nanoemulsions post dermal administration has not been fully understood. A fundamental issue is whether the integral nanoemulsions can permeate into and across the skin barriers [33]. In other words, if the nanoemulsion components along can enhance the transdermal delivery, what is the significance of the nanoemulsion structure? Furthermore, a key issue in TCI is whether, and to what extent, these nanocarriers can penetrate into the skin and deliver vaccines to APCs. However, a literature search always gives paradoxical findings. Some studies attribute enhanced dermal permeation to the penetration of integral nanoemulsions into the depth of the skin via intercellular as well as intracellular routes [34–36]. Others believe that the encapsulated drugs are released from the nanoemulsions and diffuse through the SC, while the compositions of nanoemulsions decreased the SC barriers by extracting the SC lipids as well as denaturing the proteins of keratinocytes [37–39].

This difficult situation owes much to the lack of functional approaches to identify nanoparticles against the physiological background due to the small size of the nanocarriers. Electron microscopy observation and fluorescence-based imaging are adopted to fulfil this purpose [40]. Due to similarity in compositions, it is basically impossible to discriminate nanoemulsions from dermal lipids. Although fluorescence labelling provides important information on the dermal penetration of nanoemulsions [29, 34–36, 41], the images merely demonstrate the distribution of the label which may diffuse out of the nanoemulsions while moving through the skin [33]. Furthermore, dermatopharmacokinetic analysis of the nanoemulsion components across the skin depth was adopted to answer the question [33]. However, to our best knowledge, there are still no direct evidences indicating the penetration of nanoemulsions into the skin depth. Environment-responsive probes provide excellent opportunities to discriminate the nanocarriers from signals of free probes *in vivo* [40].

Recently, our group developed a series of near-infrared (NIR) fluorescent probes with a BODIPY or aza-BODIPY structure to track the *in vivo* fate of integral nanoparticles [42–44]. The distinct feature of these probes is the sensitive aggregation-caused quenching (ACQ) effects upon contact with water through π-π stacking. These probes are highly hydrophobic and thus can be tightly embedded into either lipid or polymeric nanoparticles, where they emit intense fluorescence signals when being well dispersed. On the contrary, the fluorescence quenches immediately upon release from nanoparticles due to degradation of the matrix. These probes also display typical characteristics of the BODIPY family, e.g. high quantum yields, superior stability and pH insensitivity, which enable themselves to survive the harsh in vivo environments. In addition, since water is ubiquitous in the whole body, this on/off signal switching provides accurate and sensitive signaling to track nanoparticles throughout the whole body [40]. This rationale is also applicable to dermal conditions. Due to the ability of the skin to control water loss, water content equals to 75% of the epidermis, while even the comparatively dry SC contains 15% water [45]. The occlusive effects from the nanoemulsion film on the surface can further improve the skin hydration [46].

In this study, the dermal penetration features of nanoemulsions with particle size of 80 (NE-80), 200 (NE-200) and 500 (NE-500) nm are studied, respectively, by adopting the ACQ probe, P4 (Supplementary Figure 1), to track their *in vivo* behaviours. DAPI and immunofluorescence were used to stain normal cells and APCs for confocal laser scanning microscope (CLSM) observation, respectively. Specific attention is paid to collect evidence on the translocation of integral nanoemulsions across the skin as well as the potential of ingestion of integral nanoemulsions by APCs in skin.

## RESULTS AND DISCUSSION

### Preparation of nanoemulsions

**Figure 1:**
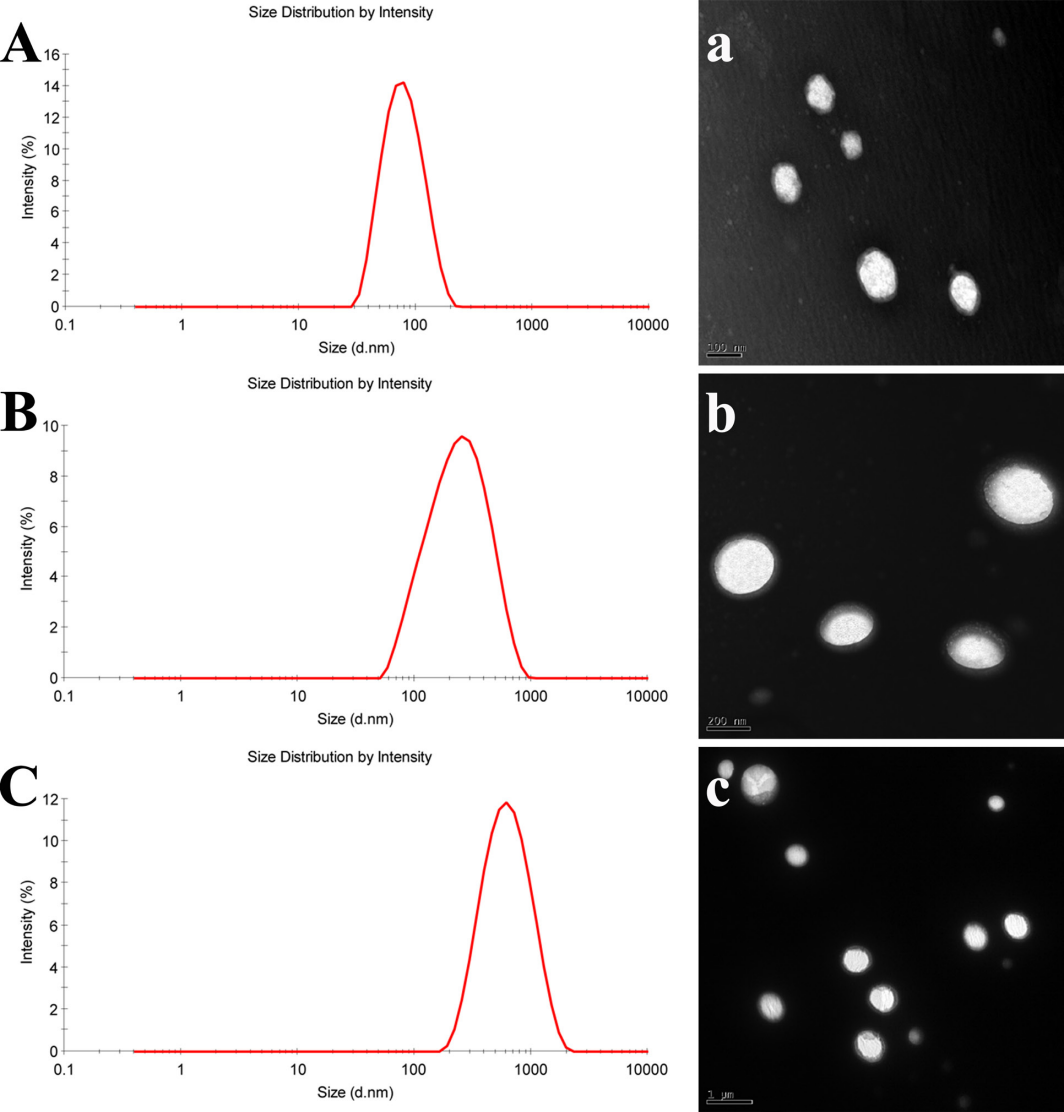
Particle size distribution and morphology of nanoemulsions **A** and **a**. NE-80; **B** and **b**. NE-200; **C** and **c**. NE-500.

Since it is accepted that particle size plays a key role in *in vivo* performance of nanoparticles, nanoemulsions with mean particle size of 80, 200 and 500 nm were prepared, respectively (Figure 1A, 1B and 1C). The PDIs are all less than 0.25, indicating a narrow size distribution. As observed by TEM, all of the nanoemulsions are spherical with smooth surfaces (Figure 1A, 1B and 1C). The observed particle sizes are coincident with the results measured by Zetasizer.

### Stability

The stability of the nanoemulsions in different medium are shown in Supplementary Figure 2. The normalized fluorescent intensity fluctuates around 100% irrespective of the particle size of the nanoemulsions and the type of the medium (Supplementary Figure 2), indicating negligible leakage of the loaded P4 probe. The reason for this is mainly attributed to the high hydrophobicity of P4 molecules. Besides, the particle size of NE-80 and NE-200 is kept unchanged during the incubation of 48 h (Supplementary Figure 2). But due to the larger size of NE-500, variation on particle size of NE-500 during incubation is a little bit higher than the smaller ones (Supplementary Figure 2).

### *In vivo* transdermal penetration

To visualize the overall transdermal translocation of nanoemulsions, DAPI was used to stain nucleus of dermal cells. It should be noted that the outermost edge of the stained tissue represents the viable epidermis instead of the SC, because SC is composed of dead cells without nucleus. During the whole experimental period, almost no fluorescence signals can be observed from skin samples treated by P4 quenched solution except that only faint fluorescence signals were observed on the surface of the viable epidermis, e.g. the SC, at 36 h (Supplementary Figure 3). Since SC is full of lipids and P4 molecule is highly hydrophobic, the rekindling of the fluorescence is mainly ascribed to the dissolve of P4 in the lipids of SC. Thus the fluorescence only appeared in the layer of the SC. This indicates the rationality of using ACQ probes, e.g. P4, to track the transdermal delivery of nanoemulsions.

### Vertical sections

**Figure 2:**
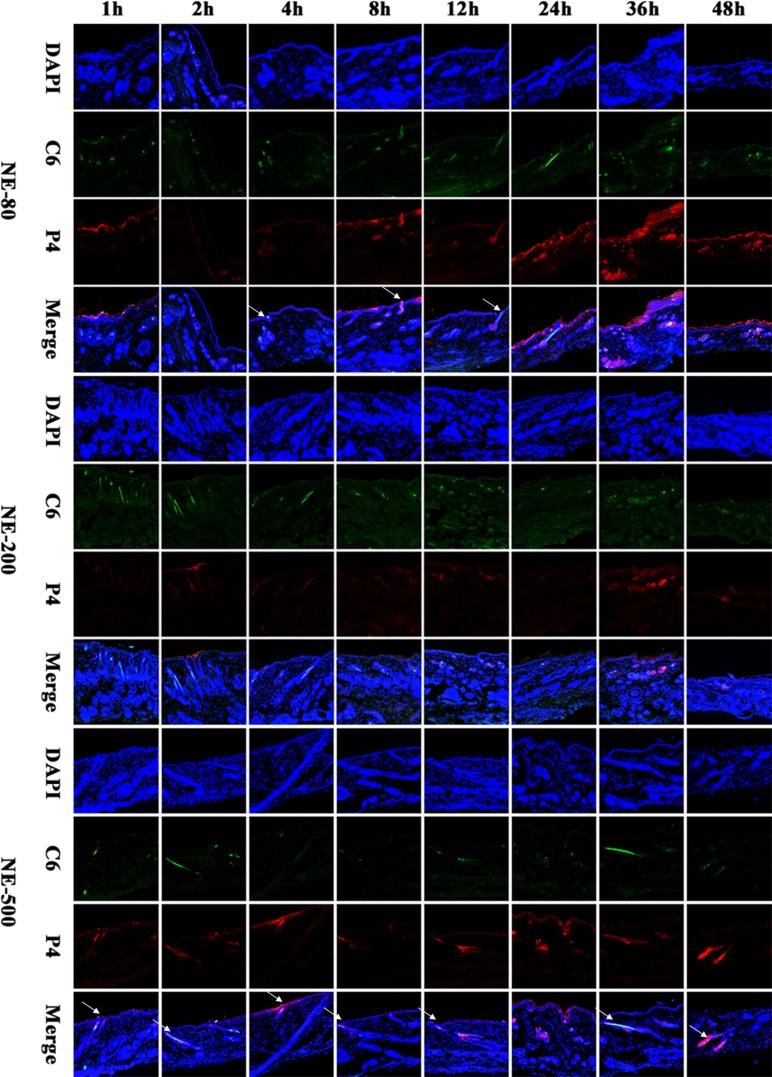
CLSM images of vertical section of the skin treated with nanoemulsions post administration (×10) The slides were stained with DAPI. White arrows indicate the hair follicles.

As observed from the vertical sections, none of the nanoemulsions could efficiently penetrate across the intact skin (Figure 2). The red fluorescence of NE-500 only appeared on the surface of the viable epidermis during the whole experimental period, indicating that NE-500 cannot penetrate the SC (Figure 2). Even though, the fluorescence intensity is faint, which may be ascribed to the poor permeability of larger nanoemulsions. However, although being retained in the SC in the first 4 h post administration, NE-80 started to permeate into the viable epidermis from 8 h post administration (Figure 2). Nonetheless, NE-80 cannot permeate into the dermis all along. At 36 h post administration, only a diffusion gradient can be observed in the viable epidermis (Figure 2), indicating that the amount of nanoemulsions that enter into the viable epidermis is still limited. It is suggested that nanoemulsions are disintegrating upon penetrating through the SC, while the components penetrate the SC with different extents and rates [33, 47]. Since the components may increase the fluidity of the SC lipids, they facilitate dermal permeation [48]. Therefore, up to 8 h post administration, small amount of NE-80 that survive the SC start to enter into the viable epidermis. However, at 48 h post administration, the fluorescent intensity in the viable epidermis is reduced to negligible levels due to degradation and/or absorption of the penetrated nanoemulsions; and the fluorescence is mainly found on the surface of the viable epidermis. The transdermal efficiency of NE-200 is at the average level among the three nanoemulsions. NE-200 enter into the viable epidermis at 36 h post administration, as indicated by the weak P4 fluorescence (Figure 2). But the colocalized regions present blue fluorescence as observed in the merged channel, indicating that the permeated amount of NE-200 is very limited.

In comparison with the poor penetration across epidermis, it is easier for nanoemulsions to enter into the hair follicles (Figure 2). NE-80 was found filling the whole hair follicles from the openings to the roots, indicating excellent trans-follicular permeation. On the contrary, NE-500 cannot efficiently permeate into the whole hair follicles, but migrate along the hair follicles to the deeper site along with time (Figure 2). At the first 24 h of the experiment, the fluorescence signals can only be observed in the upper parts of the hair follicles. Until 36 h, NE-500 permeate into the whole hair follicles, while the nanoemulsions are found depositing in the root of the hair follicles at 48 h. Since the nanoemulsions are not monodispersed, the trans-follicular transportation is mainly ascribed to the small size population in NE-500.

Coumarin 6 (C6), emitting green fluorescence, was used to mimic the cargos delivered by the nanoemulsions which emits red fluorescence. Therefore, the distribution of the green fluorescence is the same with that of the nanoemulsions. But interestingly, the penetration depth of the green fluorescence is deeper than the red fluorescence, which is ascribed to the release of C6 from nanoemulsions and the diffusion of the C6 molecules. And the components of the nanoemulsions facilitate the diffusion [47]. It should be noted that the thermodynamic activity of the cargos in the nanoemulsions is the driving force for the release and penetration of the molecules into the skin [49]. The thermodynamic activity varies in accordance with the physicochemical properties of the cargo molecules. Results from C6 cannot be expanded to all drug molecules. In this regard, nanoemulsions may be advantageous for treatment of dermal diseases instead of systemic diseases that require transdermal delivery.

### Horizontal sections

**Figure 3:**
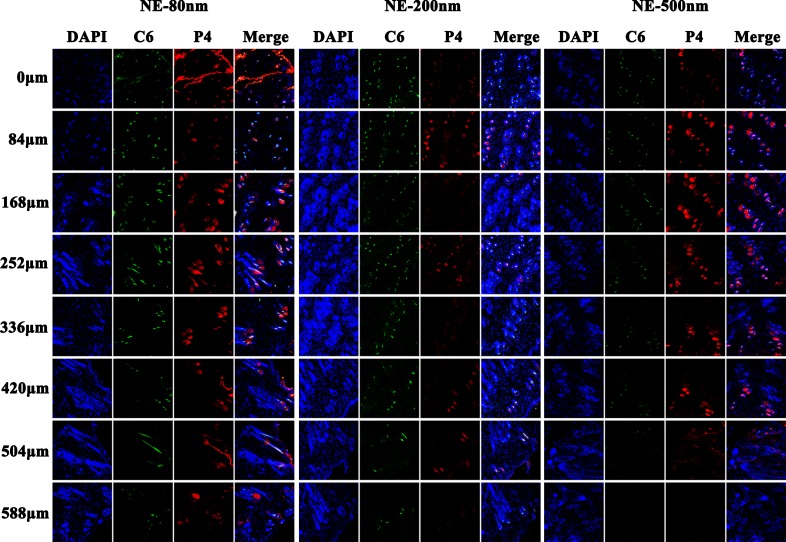
CLSM images of horizontal section of skins treated by nanoemulsion 24 h post administration (×10) The slides were stained with DAPI.

In order to further characterize the penetration depth of the nanoemulsions, continuous horizontal sections of skin samples 24 h (Figure 3) and 48 h (Supplementary Figure 4) post administration were performed, respectively. The results obtained from the horizontal sections corroborate that from the vertical sections. Ideally, the skin samples shall be flat and parallel to the section plane. But they provided curved surfaces actually. Therefore, fluorescence signals observed in the first slide of NE-80 treated skin samples present like contour lines, instead of a plane. Due to the same reason, fluorescence “contour lines” can be seen in the edge of some slides at deeper locations, which actually come from the viable epidermis. In addition, the fluorescent signals are scattered as circles in the subsequent slides of deeper sites instead of contiguous. The fluorescent areas are attributed to the hair follicles, indicating that the nanoemulsions cannot penetrate across the whole skin tissues but through the hair follicles. Nonetheless, the trans-follicular permeation of NE-80 is superior to both NE-200 and NE-500. NE-80 can reach the depth of 588 μm below the surface of the skin, whereas the other two can only reach the maximum of 504 μm. Besides, as observed in the slides of 48 h, both NE-200 and NE-500 deposited in segments instead of the whole hair follicle (Supplementary Figure 4).

### TCI by nanoemulsions

**Figure 4:**
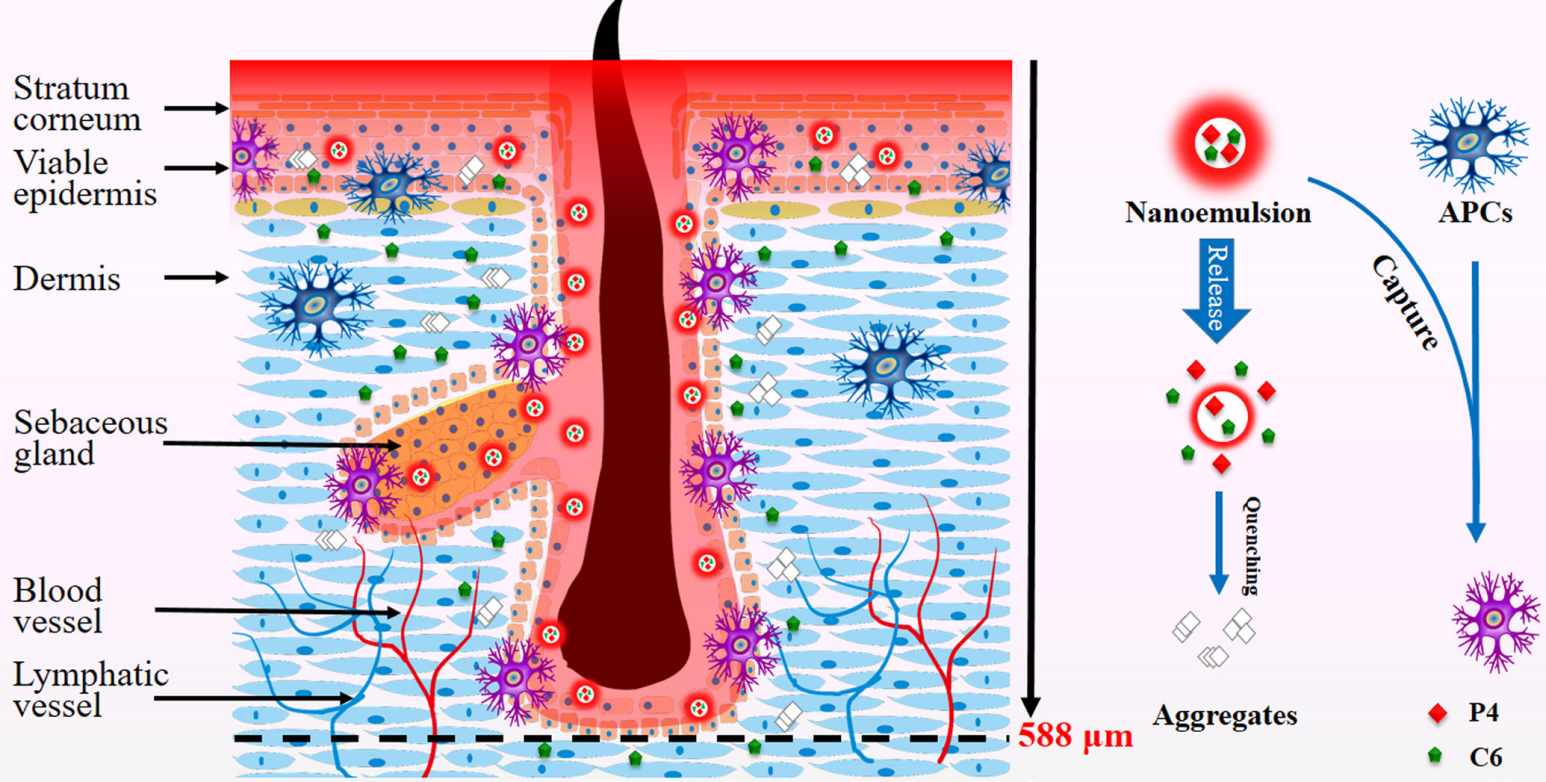
Schematic presentation of penetration of nanoemulsions into the viable epidermis and accumulation in the peri-follicular sites, implying capturing by APCs as well as potential application in TCI

Besides delivery of chemical drugs, nanoemulsions that penetrate into the viable epidermis and accumulate in hair follicles may be potent for TCI due to capture by APCs (Figure 4). Epidermis contains keratinocytes and Langerhans cells. Keratinocytes play a major immunological role as well by producing cytokins, chemokines and antimicrobial peptides in response to challenges [50]. They also belong to APCs. Langerhans cells comprise 1-3% of epidermis cells though, they cover nearly 20-25 % of the surface area due to the meshwork structure [17, 51], allowing them to uptake antigens that they encounter. The trans-follicular route has long been negligible, because hair follicles cover only 0.1% of the skin surface. However, the hair follicle is a promising target for TCI without compromising the skin barrier [21], which was firstly proved by the differences between hairy and nude mice on immune response to topical application of DNA vaccines [52]. The hair follicle provides both a rich pool of peri-follicular APCs and a permeable sites due to the absence of a SC barrier in the lower follicular orifice, facilitating antigen uptake (Figure 4) [20]. Recently, polymeric nanoparticles, smaller than 200 nm, have been found penetrating into the hair follicles and being captured by peri-follicular APCs [21–24]. But the skin should be pretreated with cyanoacrylate stripping to reduce the barrier due to the poor skin penetration of polymeric nanoparticles. Similarly, nanoemulsions that reside in epidermis and peri-follicular sites may be captured by APCs and are potent for TCI (Figure 4). Therefore, APCs are labeled by immunofluorescence staining to evaluate the potential interaction with nanoemulsions.

### Vertical sections

**Figure 5:**
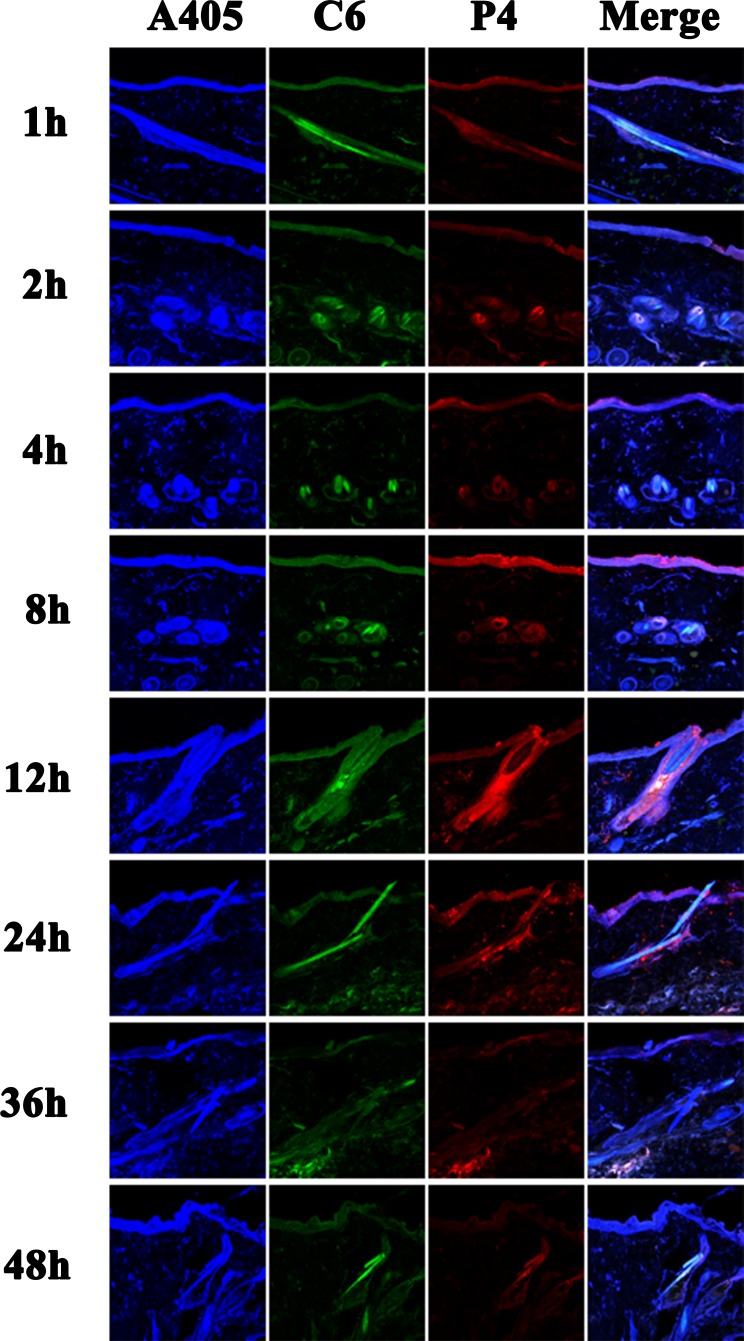
CLSM images of vertical section of skin treated by NE-80 post administration (× 20) The slides were stained with immunofluorescence.

The vertical sections of skins treated by nanoemulsions post administration are observed using CLSM under magnification folds of 20 × (Figure 5 and Supplementary Figure 5). APCs mainly reside among the keratinocytes and the peri-follicular sites. Therefore, these areas are stained as blue. It should be noted that the circular blue regions in the slides are attributed to the hair follicles. Since NE-80 is superior in dermal permeation, the red, green and blue signals overlap in these areas and show pinkish blue. It is also observed that the pinkish blue colour deepens gradually along with time and turns into deep blue at around 8 - 24 h (Figure 5). An intact hair follicle is captured in the slide of 12 h, which is full of red fluorescence of NE-80 (Figure 5). However, only the peri-follicular sites are stained to pinkish blue. All of these results indicate that NE-80 may be taken up by APCs that reside in the keratinocytes layer and the peri-follicular sites. On the contrary, almost no co-localization sites are found between NE-200/NE-500 and the APCs both in the keratinocytes layer and the peri-follicular sites (Supplementary Figure 5). Although the small size population in NE-200/NE-500 do penetrate into the hair follicles, the amount is very limited. Thus, even if the penetrated nanoemulsions were captured by the peri- follicular APCs, the colour change is negligible.

### Horizontal sections

**Figure 6:**
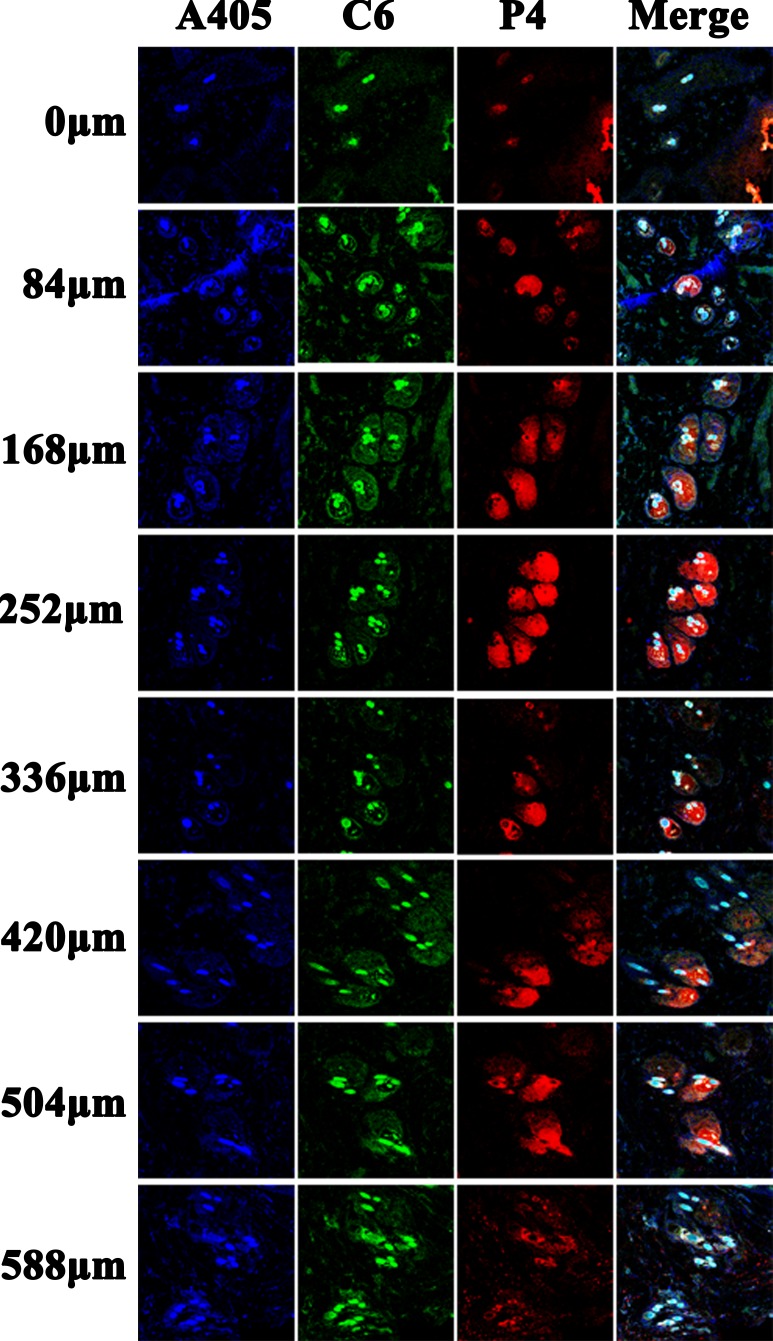
CLSM images of horizontal section of NE-80 treated skin 24 post administration (× 20) The slides were stained with immunofluorescence.

Similarly, continuous horizontal sections on skin samples 24 h (Figure 6 and Supplementary Figure 6) post administration were performed. Still, the red fluorescence observed in the slides is scattered in circular regions (Figure 6), indicating that the nanoemulsions enter into the skin mainly by trans-follicular route instead of trans-epidermal route. Faint green signals can also be found in the surrounding areas outside the circular regions, because of the diffusion of C6 molecules that are released from the nanoemulsions. Nonetheless, NE-80 show superior trans-follicular efficiency to NE-200/NE-500. NE-80 show stronger fluorescence intensity than the other two nanoemulsions with deeper penetration depth to 588 μm (Figure 6 and Supplementary Figure 6). Moreover, the edge of the hair follicles are stained by pink blue as observed in the merged channel for skin samples treated by NE-80, implying the possible capture of nanoemulsions by the peri-follicular APCs. However, concerning the NE-200/NE-500 treated skin samples, the red fluorescent signals from the nanoemulsions are mainly surrounded by the blue signals from the peri-follicular APCs (Supplementary Figure 6). The reason for this phenomenon is primarily ascribed to the less amount of NE-200/NE-500 accumulated in the hair follicles. Although the nanoemulsions can be captured by the peri-follicular APCs, it cannot lead to obvious colour change in the merged channel.

In summary, the ACQ probe, P4, can be used to track the transdermal translocation of nanoemulsions. Nanoemulsions can diffuse into but not efficiently penetrate the viable epidermis. The trans-follicular route is the primary route that nanoemulsions permeate into the deep sites of the skin. The cargos can be released from the nanoemulsions and diffuse into the surrounding dermal tissues where nanoemulsions reside. Particle size plays a significant role in the dermal translocation of nanoemulsions. Small nanoemulsions, such as 80 nm, can permeate into the viable epidermis as well as fill in the whole hair follicles, whereas bigger nanoemulsions, such as 500 nm, cannot efficiently penetrate the SC and only migrate along the hair follicles. Nanoemulsions with median size, such as 200 nm, exhibit moderate transdermal delivery effects among the three-size nanoemulsions. Furthermore, co-localization between nanoemulsions and APCs in the viable epidermis and peri-follicular sites implies possible capture of nanoemulsions by APCs. In conclusion, nanoemulsions, especially smaller than 200 nm, are advantageous for dermal drug delivery and potential in transcutaneous immunization.

## MATERIALS AND METHODS

### Materials

Water-quenching NIR fluorescent probe P4 (λ_abs_/λ_em_ = 651/662) was synthesized in our group. Kolliphor^®^ HS 15 was kindly provided by BASF Advanced Chemicals Co., Ltd, China. Labrafac Lipophile WL 1349 was a gift from Gattefossé Co., Cedex, France. Normal Donkey Serum (Jackson Immuno Research Laboratories, Inc., USA), Anti-CD1a antibody, Donkey Anti-Mouse IgG H&L (Abcam. Cambridge, UK) and Image-iT™ FX Signal Enhancer (Molecular Probes, Inc., USA) were purchased from a local agency. C6 was from Sigma-Aldrich, St Louis, USA. 4′,6-diamidino-2-phenylindole (DAPI) was from Yeasen Bio-tech Co., Ltd, Shanghai, China. 4% paraformaldehyde (PFA) was from Fortune bio-tech Co., Ltd, Shanghai, China. OCT compounds were from Leica, Germany. Deionized water was prepared by using a Milli-Q purification system (Millipore, USA). Other reagents were of analytical grade and purchased from local distributors.

### Preparation of nanoemulsions

P4 dichloromethane solution (40 μg/mL), 2 mL, and C6 dichloromethane solution (100 μg/mL), 1 mL, was mixed with 5.0 g Lipophile WL 1349. The solution was kept at 40°C and blew with nitrogen to remove dichloromethane to form the oil phase. The aqueous phase was a solution of Kolliphor^®^ HS 15 (3.0 g dissolved in 35 mL water). Under magnetic stirring, the oil phase was instilled into the aqueous phase and mixed for 10 min. The mixture was emulsified by high-speed shearing (Scientz Biotechnology Co., Ltd., China) under 10000 rpm for 10 s to form the crude emulsion. The crude emulsion was subjected to different processes to obtain nanoemulsions of different particle sizes. Nanoemulsions of 80 nm (NE-80) and 200 nm (NE-200) were prepared by homogenization (AH 100 D; ATS Engineering Inc, Brampton, ON, Canada) under 1000 bar for 3 min and 120 bar for 2 min, respectively. Membrane emulsifier (Senhui Microsphere Technology (Suzhou) Co., Ltd., China) was used to prepare nanoemulsions of 500 nm (NE-500) by sequentially extruding the crude emulsions through a membrane tube with a pore size of 1.2 μm at 1.6 MPa for 4 cycles and 0.8 μm at 1.8 MPa for 2 cycles.

### Characterization of nanoemulsions

#### Particle size

The mean particle size and polydispersity index (PDI) of nanoemulsions were measured using a Malvern Zetasizer Nano^®^ (Malvern Instruments, Malvern UK) with a 4 mW He-Ne laser at 633 nm under ambient temperature. Prior to the measurement, the samples were diluted by 10 folds using Milli-Q water and balanced for 120 s in the instrument. Triplicate measurements were performed for each sample.

### Morphology

Transmission electron microscopy (TEM) (Jeol JEM 2100F, Japan) was used to observe the morphology of the nanoemulsions. The samples were diluted by 50 folds using Milli-Q water, which was stained by mixing with equal volume of 1 % uranyl acetate solution. Then the solution was applied on carbon-coated grid for TEM observation.

### Stability

The stability of the nanoemulsions in hydrochloric acid solution (HCl) (pH 1.2), acetate buffer solution (ABS) (pH 4.5), phosphate buffer solution (PBS) (pH 6.8 and 7.4), 1% sodium dodecyl sulfate (SDS) (w/v) and 2% tween 80 (w/v) aqueous solution was evaluated, respectively. During the experiment, 1 mL nanoemulsions were mixed with 10 mL media and incubated in a water bath at 37±0.5°C. Before and 1, 2, 4, 8, 12, 24, 36 and 48 h post incubation, samples were withdrawn to measure the fluorescence intensity and the particle size. The fluorescent intensity was measured by Cary Eclipse spectofluorometer (Agilent Technologies, Inc. US) at an excitation wavelength of 651 nm and an emission wavelength of 662 nm.

### *In vivo* skin permeation studies

The experiments were approved by the institutional ethical committee and performed in compliance with the institutional guidelines at School of Pharmacy, Fudan University. SD rats (Male, 180-200 g) were raised in rooms controlled at 23 ± 1°C and 55 ± 5% relative humidity as well as 12 h light/12 h dark time cycles. Standard laboratory chow diet and tap water were provided during acclimatization. The abdominal hair of the rats were removed using depilator 12 h prior to the experiment. The rats were anaesthetized by intraperitoneal injection of 10% chloral hydrate aqueous solution and fixed by a rat fixator. For ease of drug administration, the donor cell of the Franz diffusion cells was fixed by super glue on the abdominal surface. The internal diameter of the cell is 10 mm. Nanoemulsions, 200 μL, were applied to the skin through the open cap of the donor cell. The rats were sacrificed at 1, 2, 4, 8, 12, 24, 36 and 48 h post administration. The remaining formulation was cleaned with saline. The investigational skin was carefully excised and stored at -80°C.

### Frozen section

The skins were split into four equal blocks. The skins were embedded in the OCT compound. Both horizontal and vertical cryosections of 7 μm were performed. For vertical section, cutting was performed from dermis towards SC to avoid artefacts of nanoemulsions translocation. Continuous horizontal sections were performed, starting from SC, one slice out of every 12 sections was collected. These slices were further subjected to either DAPI or immunofluorescence staining.

### DAPI staining

The slides were rinsed sequentially with pure water twice (5 min each) and pH 7.4 PBS thrice (5 min each). PFA, 4 %, was instilled on the surface of the slide and incubated for 10 min at room temperature. After this, PFA was removed by washing with pH 7.4 PBS. Then the slide was stained by DAPI solution, 5 μg/mL, for 20 min. After removal of DAPI, the slide was mounted by buffered glycerol.

### Immunofluorescence staining

The process to the slide in immunofluorescence staining is the same as DAPI staining, except that the slide was put in citrate buffer solution at 95°C for 20 min, following removal of PFA. After three washes in pH 7.4 PBS, the slide was mounted with donkey serum and incubated for 30 min at room temperature. Following removal of the serum with three washes in pH 7.4 PBS, the slide was incubated with 0.5% Tween-20 solution for 20 min. Then, signal enhancer was introduced to the slide and incubated for 30 min. The slide was further incubated for 4 h at room temperature with Anti-CD1a antibody and another 1 h with Donkey Anti-Mouse IgG H&L. Finally, the slide was mounted by buffered glycerol.

### Confocal laser scanning microscope

The slides were visualized with Zeiss LSM 710 confocal laser scanning microscope (CLSM) (Carl Zeiss Inc., Germany). Fluorescence from DAPI and C6 was excited by corresponding default channels. Immunofluorescence was excited by A405 channel. P4 signal was excited by Alexa633 channel. The resolution of the scan was set to 1024 × 1024 pixeles. Image display and analysis were performed using the software provided by the supplier.

## SUPPLEMENTARY MATERIALS FIGURES



## References

[1] Tsai MJ, Fu YS, Lin YH, Huang YB, Wu PC (2014). The effect of nanoemulsion as a carrier of hydrophilic compound for transdermal delivery. PLoS One.

[2] Alexander A, Dwivedi S, Ajazuddin Giri TK, Saraf S, Saraf S, Tripathi DK (2012). Approaches for breaking the barriers of drug permeation through transdermal drug delivery. J Control Release.

[3] Liuzzi R, Carciati A, Guido S, Caserta S (2016). Transport efficiency in transdermal drug delivery: What is the role of fluid microstructure?. Colloids Surf B Biointerfaces.

[4] Lee JK, Kang SM, Yang SH, Cho WK (2015). Micro/Nanostructured Films and Adhesives for Biomedical Applications. J Biomed Nanotechnol.

[5] Barry BW (2001). Novel mechanisms and devices to enable successful transdermal drug delivery. Eur J Pharm Sci.

[6] Giannos SA (2015). Identifying present challenges to reliable future transdermal drug delivery products. Ther Deliv.

[7] Kumar S, Zakrewsky M, Chen M, Menegatti S, Muraski JA, Mitragotri S (2015). Peptides as skin penetration enhancers: mechanisms of action. J Control Release.

[8] Pham QD, Bjorklund S, Engblom J, Topgaard D, Sparr E (2016). Chemical penetration enhancers in stratum corneum - Relation between molecular effects and barrier function. J Control Release.

[9] Lane ME (2013). Skin penetration enhancers. Int J Pharm.

[10] Lauterbach A, Muller-Goymann CC (2015). Applications and limitations of lipid nanoparticles in dermal and transdermal drug delivery via the follicular route. Eur J Pharm Biopharm.

[11] Vogt A, Wischke C, Neffe AT, Ma N, Alexiev U, Lendlein A (2016). Nanocarriers for drug delivery into and through the skin - Do existing technologies match clinical challenges?. J Control Release.

[12] Zhang YT, Han MQ, Shen LN, Zhao JH, Feng NP (2015). Solid Lipid Nanoparticles Formulated for Transdermal Aconitine Administration and Evaluated *In Vitro* and *In Vivo*. J Biomed Nanotechnol.

[13] Chen YZ, Huang YK, Chen Y, Ye YJ, Lou KY, Gao F (2015). Novel nanoparticles composed of chitosan and beta-cyclodextrin derivatives as potential insoluble drug carrier. Chinese Chem Lett.

[14] Fang JY, Leu YL (2006). Prodrug strategy for enhancing drug delivery via skin. Curr Drug Discov Technol.

[15] Sloan KB, Devarajan-Ketha H, Wasdo SC (2011). Dermal and transdermal delivery: prodrugs. Ther Deliv.

[16] Mitragotri S (2005). Immunization without needles. Nat Rev Immunol.

[17] Mishra DK, Dhote V, Mishra PK (2013). Transdermal immunization: biological framework and translational perspectives. Expert Opin Drug Deliv.

[18] Gill HS, Kang SM, Quan FS, Compans RW (2014). Cutaneous immunization: an evolving paradigm in influenza vaccines. Expert Opin Drug Deliv.

[19] Hirobe S, Okada N, Nakagawa S (2013). Transcutaneous vaccines—current and emerging strategies. Expert Opin Drug Deliv.

[20] Mittal A, Raber AS, Lehr CM, Hansen S (2013). Particle based vaccine formulations for transcutaneous immunization. Hum Vaccin Immunother.

[21] Mittal A, Raber AS, Schaefer UF, Weissmann S, Ebensen T, Schulze K, Guzman CA, Lehr CM, Hansen S (2013). Non-invasive delivery of nanoparticles to hair follicles: a perspective for transcutaneous immunization. Vaccine.

[22] Rancan F, Amselgruber S, Hadam S, Munier S, Pavot V, Verrier B, Hackbarth S, Combadiere B, Blume-Peytavi U, Vogt A (2014). Particle-based transcutaneous administration of HIV-1 p24 protein to human skin explants and targeting of epidermal antigen presenting cells. J Control Release.

[23] Vogt A, Hadam S, Deckert I, Schmidt J, Stroux A, Afraz Z, Rancan F, Lademann J, Combadiere B, Blume-Peytavi U (2015). Hair follicle targeting, penetration enhancement and Langerhans cell activation make cyanoacrylate skin surface stripping a promising delivery technique for transcutaneous immunization with large molecules and particle-based vaccines. Exp Dermatol.

[24] Mittal A, Schulze K, Ebensen T, Weissmann S, Hansen S, Lehr CM, Guzman CA (2015). Efficient nanoparticle-mediated needle-free transcutaneous vaccination via hair follicles requires adjuvantation. Nanomedicine.

[25] Lin XY, Bai G, Sutherland K, Costanza F, Breitenkamp K, Sill K, Cai JF, Cao CH (2016). Polymer-Encapsulated A beta Peptide Fragments as an Oligomeric-Specific Vaccine for Alzheimer's Disease. J Biomed Nanotechnol.

[26] Bernardi DS, Bitencourt C, da Silveira DS, da Cruz EL, Pereira-da-Silva MA, Faccioli LH, Lopez RF (2016). Effective transcutaneous immunization using a combination of iontophoresis and nanoparticles. Nanomedicine.

[27] Lu Y, Qi J, Wu W (2012). Absorption, disposition and pharmacokinetics of Nanoemulsions. Curr Drug Metab.

[28] McClements DJ (2012). Nanoemulsions versus microemulsions: terminology, differences, and similarities. Soft Matter.

[29] Choi S, Kim JW, Lee YJ, Delmas T, Kim C, Park S, Lee H (2014). Evaluation of transdermal delivery of nanoemulsions in ex vivo porcine skin using two-photon microscopy and confocal laser-scanning microscopy. J Biomed Opt.

[30] Moghadam SH, Saliaj E, Wettig SD, Dong C, Ivanova MV, Huzil JT, Foldvari M (2013). Effect of chemical permeation enhancers on stratum corneum barrier lipid organizational structure and interferon alpha permeability. Mol Pharm.

[31] Bouchemal K, Briançon S, Perrier E, Fessi H (2004). Nano-emulsion formulation using spontaneous emulsification: solvent, oil and surfactant optimisation. Int J Pharm.

[32] Gogoll K, Stein P, Lee KD, Arnold P, Peters T, Schild H, Radsak M, Langguth P (2016). Solid nanoemulsion as antigen and immunopotentiator carrier for transcutaneous immunization. Cell Immunol.

[33] Hathout RM, Mansour S, Geneidi AS, Mortada ND (2011). Visualization, dermatopharmacokinetic analysis and monitoring the conformational effects of a microemulsion formulation in the skin stratum corneum. J Colloid Interface.

[34] Khurana S, Jain NK, Bedi PM (2013). Nanoemulsion based gel for transdermal delivery of meloxicam: physico-chemical, mechanistic investigation. Life Sci.

[35] Somagoni J, Boakye CH, Godugu C, Patel AR, Mendonca Faria HA, Zucolotto V, Singh M (2014). Nanomiemgel—a novel drug delivery system for topical application—in vitro and in vivo evaluation. PLoS One.

[36] Kim JH, Ko JA, Kim JT, Cha DS, Cho JH, Park HJ, Shin GH (2014). Preparation of a capsaicin-loaded nanoemulsion for improving skin penetration. J Agr Food Chem.

[37] Shakeel F, Baboota S, Ahuja A, All J, Shafiq S (2008). Skin permeation mechanism of aceclofenac using novel nanoemulsion formulation. Pharmazie.

[38] Lee PJ, Langer R, Shastri VP (2003). Novel microemulsion enhancer formulation for simultaneous transdermal delivery of hydrophilic and hydrophobic drugs. Pharm Res.

[39] Kogan A, Garti N (2006). Microemulsions as transdermal drug delivery vehicles. Adv Colloid Interface Sci.

[40] Hu X, Dong X, Lu Y, Qi J, Zhao W, Wu W (2017). Bioimaging of nanoparticles: the crucial role of discriminating nanoparticles from free probes. Drug Discov Today.

[41] Yu M, Ma H, Lei M, Li N, Tan F (2014). In vitro/in vivo characterization of nanoemulsion formulation of metronidazole with improved skin targeting and anti-rosacea properties. Eur J Pharm Biopharm.

[42] Hu X, Fan W, Yu Z, Lu Y, Qi J, Zhang J, Dong X, Zhao W, Wu W (2016). Evidence does not support absorption of intact solid lipid nanoparticles via oral delivery. Nanoscale.

[43] Xie Y, Hu X, He H, Xia F, Ma Y, Qi J, Dong X, Zhao W, Lu Y, Wu W (2016). Tracking translocation of glucan microparticles targeting M cells: implications for oral drug delivery. J Mater Chem B.

[44] Hu X, Zhang J, Yu Z, Xie Y, He H, Qi J, Dong X, Lu Y, Zhao W, Wu W (2015). Environment-responsive aza-BODIPY dyes quenching in water as potential probes to visualize the in vivo fate of lipid-based nanocarriers. Nanomedicine.

[45] Kumar A, Pathak K, Bali V (2012). Ultra-adaptable nanovesicular systems: a carrier for systemic delivery of therapeutic agents. Drug Discov Today.

[46] Ngan CL, Basri M, Tripathy M, Abedi Karjiban R, Abdul-Malek E (2015). Skin intervention of fullerene-integrated nanoemulsion in structural and collagen regeneration against skin aging. Eur J Pharm Sci.

[47] Abdel-Mottaleb MM, Neumann D, Lamprecht A (2011). Lipid nanocapsules for dermal application: a comparative study of lipid-based versus polymer-based nanocarriers. Eur J Pharm Biopharm.

[48] Ge S, Lin Y, Lu H, Li Q, He J, Chen B, Wu C, Xu Y (2014). Percutaneous delivery of econazole using microemulsion as vehicle: formulation, evaluation and vesicle-skin interaction. Int J Pharm.

[49] Kim BS, Won M, Lee KM, Kim CS (2008). In vitro permeation studies of nanoemulsions containing ketoprofen as a model drug. Drug Deliv.

[50] Nasr M, Abdel-Hamid S, Alyoussef AA (2015). A highlight on lipid based nanocarriers for transcutaneous immunization. Curr Pharm Biotechnol.

[51] Glenn GM, Kenney RT, Ellingsworth LR, Frech SA, Hammond SA, Zoeteweij JP (2003). Transcutaneous immunization and immunostimulant strategies: capitalizing on the immunocompetence of the skin. Expert Rev Vaccines.

[52] Fan H, Lin Q, Morrissey GR, Khavari PA (1999). Immunization via hair follicles by topical application of naked DNA to normal skin. Nat Biotechnol.

