# Supplementary material for: Size-dependent penetration of nanoemulsions into epidermis and hair follicles: implications for transdermal delivery and immunization
Source: Oncotarget. 2017 Apr 16;8(24):38214–26. doi: 10.18632/oncotarget.17130 (PMC5503527; doi:10.18632/oncotarget.17130)
Supplement: Supplementary file 1 [file oncotarget-08-38214-s001.pdf]

Size-dependent penetration of nanoemulsions into epidermis and hair follicles: implications for transdermal delivery and immunization

Supplementary Material

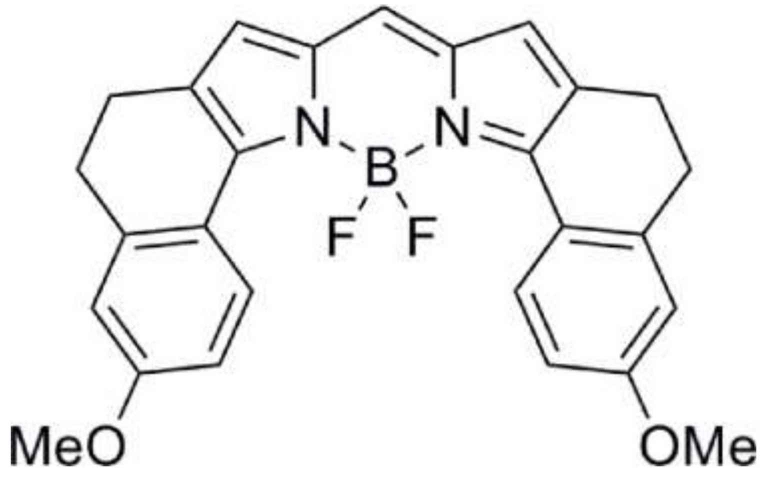

Supplementary Figure 1: Molecular structure of P4. (2Z)-7-methoxy-2-[(7-methoxy-4,5-dihydro-1H-benzo[g]indol-2-yl)methylene]-4,5-dihydrobenzo[g]indole boron difluoride.

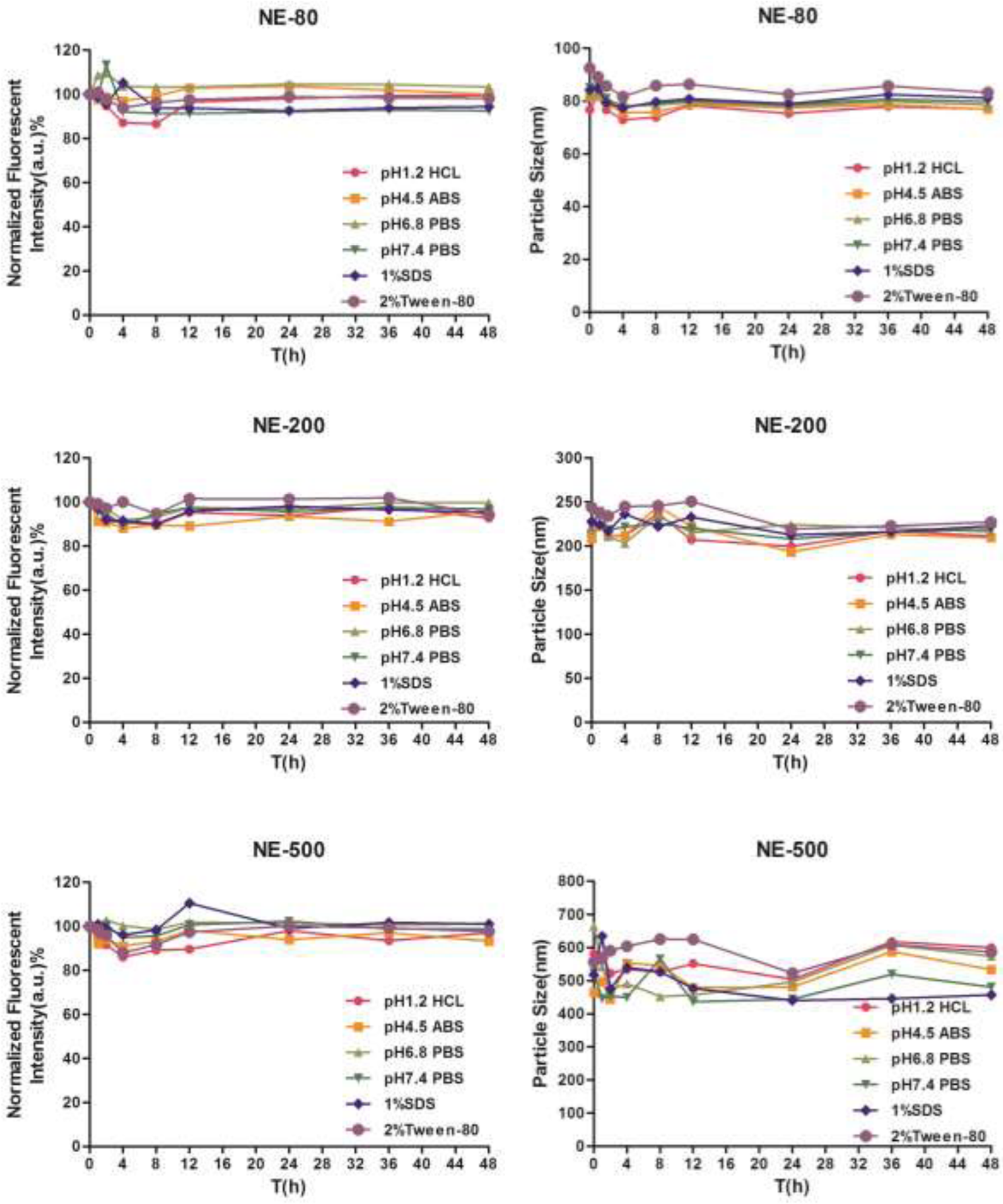

Supplementary Figure 2: Stability of nanoemulsions in different medium.

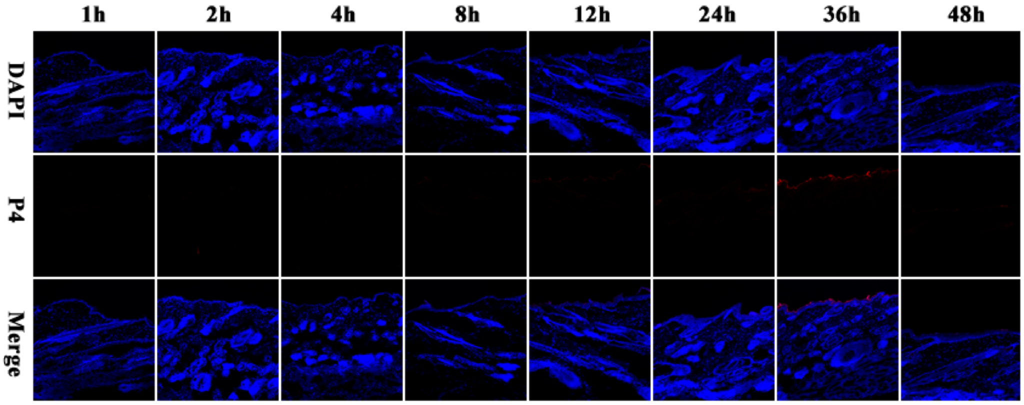

Supplementary Figure 3: CLSM images of vertical section of skin treated by P4 quenched solution post administration (×10). The slides were stained with DAPI.

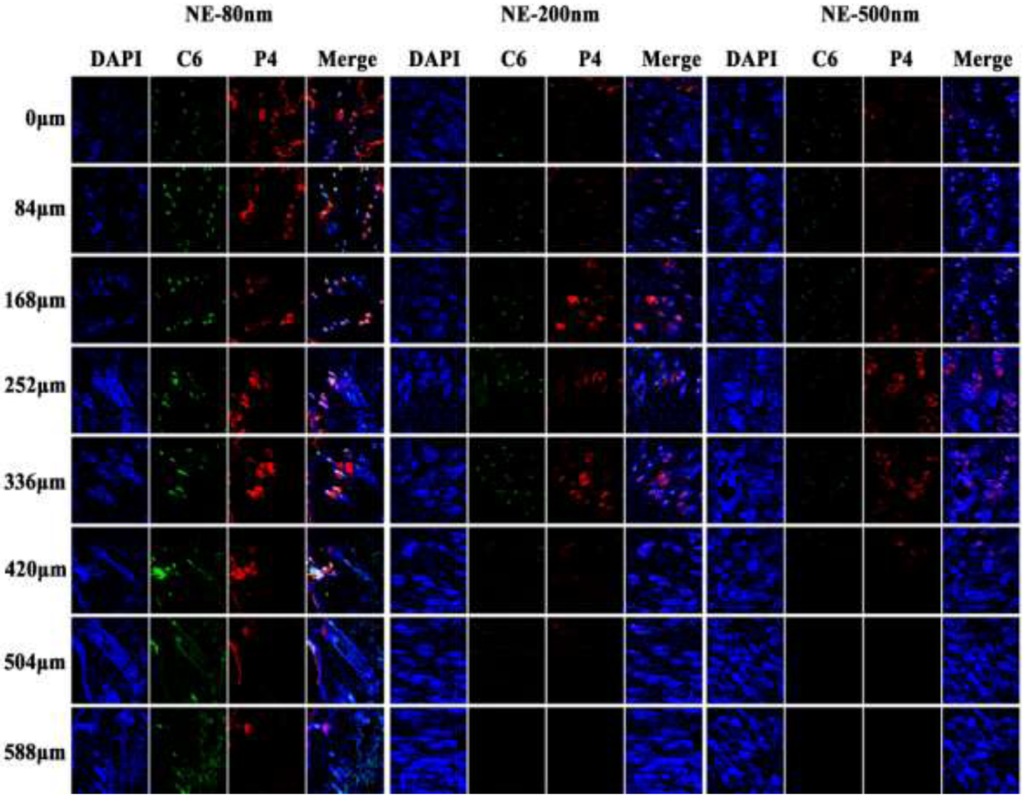

Supplementary Figure 4: CLSM images of horizontal section of skin treated by nanoemulsions 48 h post administration (×10). The slides were stained with DAPI.

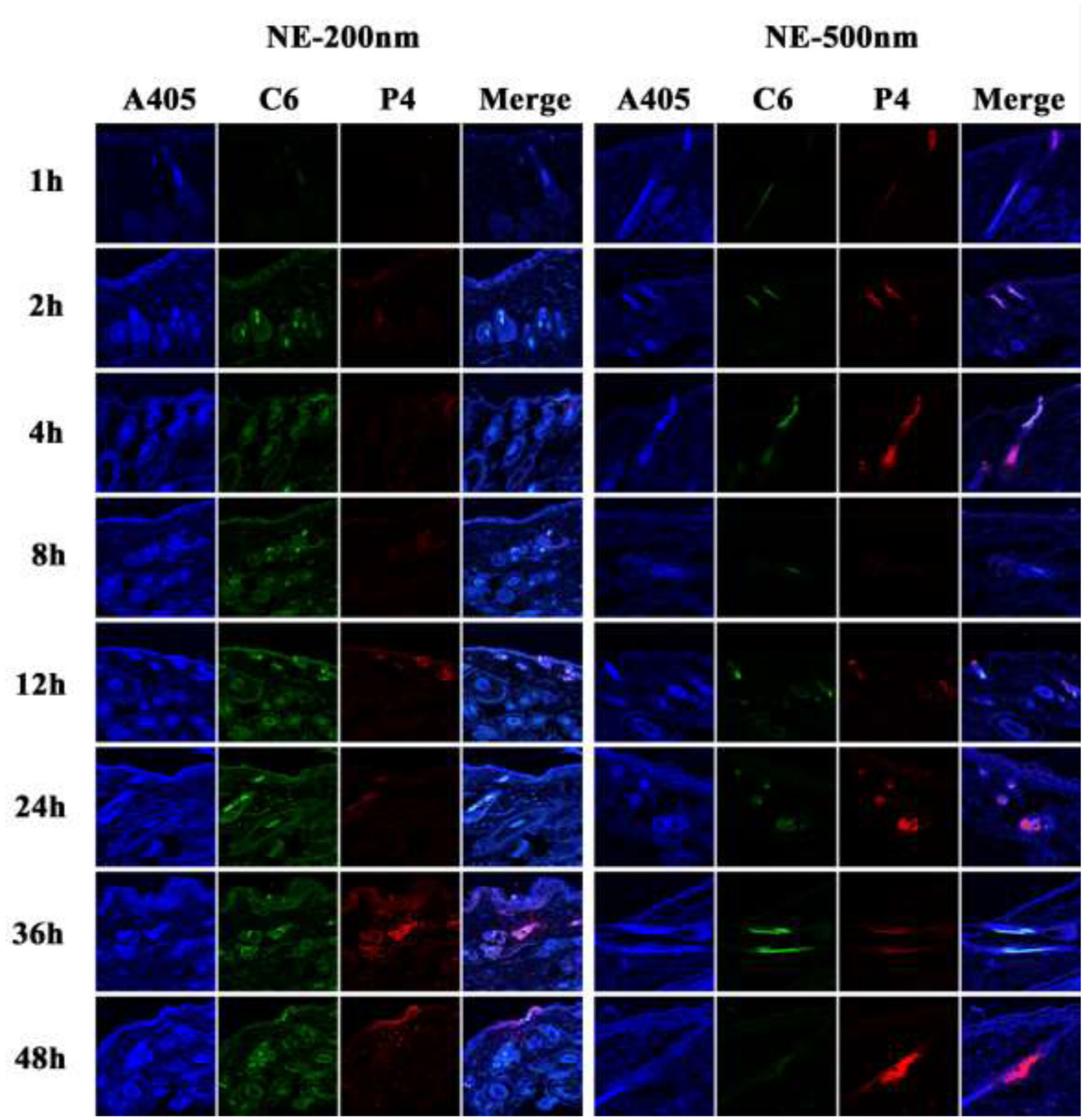

Supplementary Figure 5: CLSM images of vertical section of skin treated by NE-200 and NE-500 post administration (×20). The slides were stained with immunofluorescence.

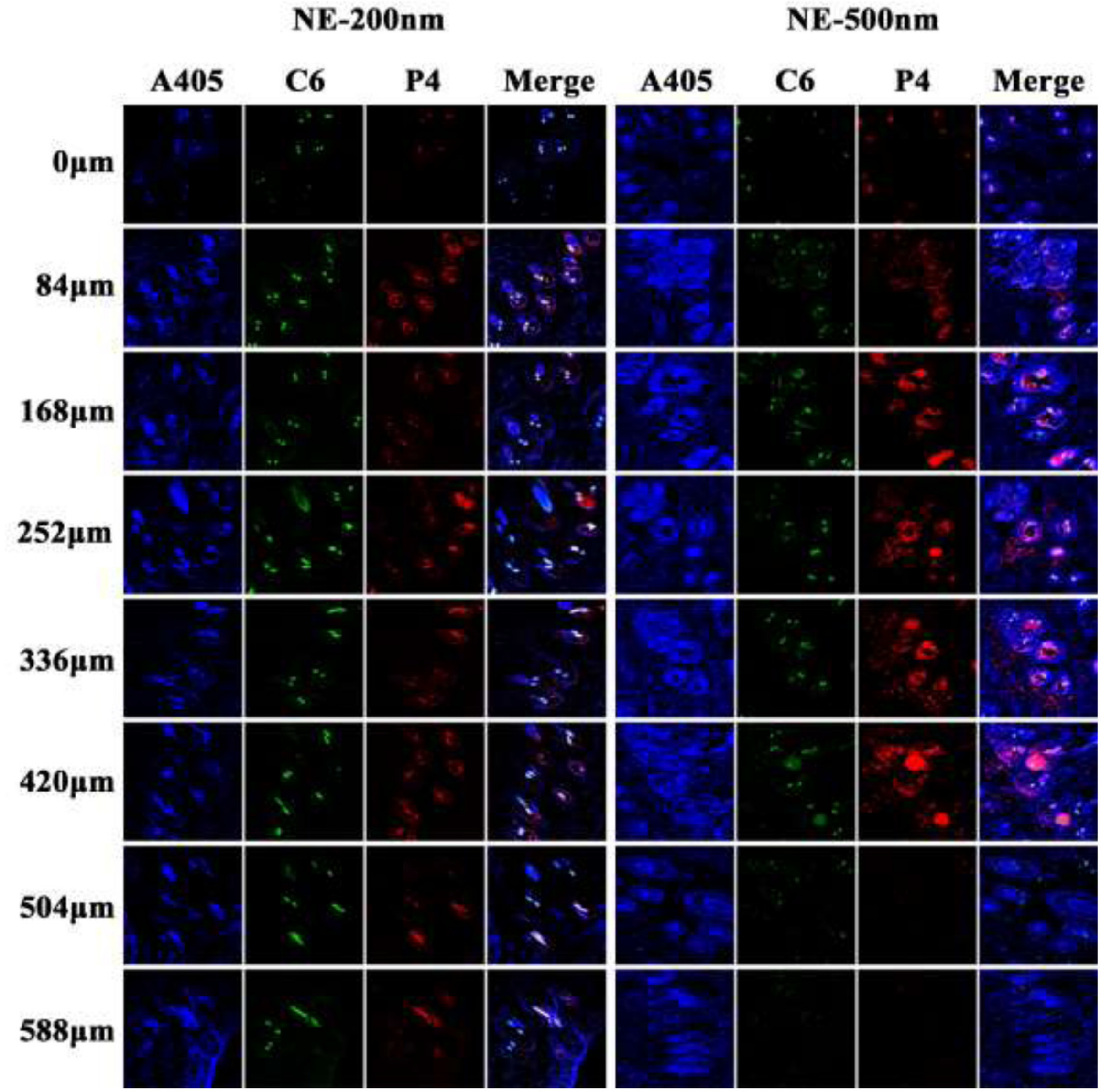

Supplementary Figure 6: CLSM images of horizontal section of skin treated by NE-200 and NE-500 24 h post administration (×20). The slides were stained with immunofluorescence.
